# Clinical and immunological markers of peanut allergy severity and tolerance in children

**DOI:** 10.3389/falgy.2026.1826635

**Published:** 2026-06-03

**Authors:** Tadej Petek, Brigita Koren, Maja Tomazin, Tina Hojnik, Vojko Berce, Mija Lajhar, Blažka Krašovec, Matjaž Homšak, Maja Skerbinjek Kavalar, Peter Korošec

**Affiliations:** 1 Department of Paediatrics, University Medical Centre Maribor, Maribor, Slovenia; 2 Faculty of Medicine, University of Maribor, Maribor, Slovenia; 3 Pediatric Allergy Outpatient Clinic, Maribor, Slovenia; 4 Laboratory for Clinical Immunology and Molecular Genetics, University Clinic of Respiratory and Allergic Diseases Golnik, Golnik, Slovenia; 5 Faculty of Pharmacy, University of Ljubljana, Ljubljana, Slovenia

**Keywords:** basophil activation test, children, component-resolved diagnostics, peanut allergy, severity, tolerance

## Abstract

**Background:**

Improved diagnostic tools are needed to assess the risk of severe peanut allergy (PA) and/or predict its resolution.

**Methods:**

This prospective multicenter cross-sectional study with follow-up elements (2020–2024) included children aged 3 months–18 years with confirmed PA. Diagnosis relied on clinical history, skin-prick test wheals (SPT), peanut sIgE, and/or oral food challenge (OFC). Children with isolated systemic cutaneous reactions (World Allergy Organization—WAO grade 1) were compared with children with WAO grade ≥2 reactions. Basophil activation testing (BAT) and component-resolved diagnostics were performed in all participants, and OFC assessed tolerance in selected children.

**Results:**

Among 51 children (24 WAO grade 1 and 27 grade 2–5), those with anaphylaxis had larger SPT wheals (*p* = 0.037) and peanut sIgE levels (*p* = 0.036). Children with anaphylaxis had greater sensitization to Ara h 1 (*p* = 0.021), Ara h 2 (*p* = 0.041), and Ara h 6 (*p* = 0.030) and higher BAT responses after adjustment for age, total IgE, and the number of food allergens to which they were allergic. Overall, receiver operating characteristic curve analyses showed area under the curve values ranging from 0.75 to 0.8 for Ara h 1, peanut sIgE, Ara h 2, Ara h 3, and Ara h 6 and SPT and BAT at greatest concentrations. OFC was performed in 20 children (6 with anaphylaxis). Tolerance was associated with lower sIgE (*p* = 0.033), Ara h 2 (*p* = 0.032) and Ara h 6 (*p* = 0.020), and BAT showed a possible association (*p* = 0.060) after adjustment for relevant confounders.

**Conclusion:**

Several biomarkers, including peanut SPT, sIgE, Ara h 1, Ara h 2, Ara h 6, and BAT showed moderate discrimination between systemic cutaneous reactions and anaphylaxis, while peanut sIgE, Ara h 2, and Ara h 6 were associated with clinical tolerance. Evaluating these biomarkers may help reduce the need for OFCs in selected cases.

## 1 Introduction

Peanut allergy (PA) is one of the most common and potentially life-threatening food allergies in children, with a rising prevalence in many parts of the world. Inaccurate diagnosis of food allergy (FA) carries significant consequences—overdiagnosis may lead to unnecessary dietary restrictions and increased allergy risk, while underdiagnosis can result in life-threatening reactions. Current diagnostic methods such as clinical history, IgE sensitization tests, and oral food challenges (OFCs) have limitations despite improvements resulting from component-resolved diagnostics (CRD) and refined cutoff values (1, 2). Novel tools such as basophil activation tests (BATs) increase the sensitivity and specificity of PA diagnosis alone and in conjunction with sIgE and skin-prick testing (3). Machine learning algorithms may support efficient allergy diagnosis, enabling personalized digitalized medicine through data-driven approaches under an appropriate legal framework (4, 5).

Several biomarkers, including skin-prick test (SPT), peanut sIgE levels, Ara h 2, and BAT, have been described as predictors of severity during OFC (6). Sensitization to Ara h 2 has been linked to a higher likelihood of systemic allergic reactions, and levels above 1.4 kU/L suggest a more severe PA phenotype (7). In children, levels ≥5.0 kU/L have been associated with clinical reactivity and ≤0.1 kU/L with tolerance (8). However, in another study, CRD (including Ara h 2) did not correlate to the severity of the reaction or the eliciting dose (9). Host factors, including comorbid conditions such as poorly controlled asthma, age, and behavioral variables including exercise, smoking, and certain medications further complicate severity prediction (7).

Despite these findings, few studies have comprehensively examined clinical and immunological profiles distinguishing mild systemic from more severe PA phenotypes in children. Understanding these differences may not only enhance risk stratification, such as the need for epinephrine administration, inpatient admission, and autoinjector prescription, but also inform decisions regarding OFCs and the timing of reevaluation for potential resolution.

In this study, we aimed to characterize the clinical features and immunological markers associated with systemic cutaneous reactions and PA-induced anaphylaxis in a well-defined pediatric cohort. We also explored the predictors of allergy resolution over time, with the goal of identifying useful clinical and immunological markers to support personalized allergy management strategies and reduce the need for OFCs.

## 2 Methods

### 2.1 Design and study population

This was a prospective multicenter cross-sectional study with follow-up elements, which assessed children with an established PA diagnosis, aged 3 months to 18 years, to determine PA severity and identify those who developed tolerance. Data were collected between 1 January 2020 and 31 December 2024. Patients were recruited from a tertiary hospital pediatric allergy clinic and a second outpatient pediatric allergy clinic. Initial diagnosis of PA was defined by the presence of an allergic reaction to peanuts within 2 h after consumption and a positive allergy test, either a peanut SPT or a peanut sIgE. In the absence of a positive allergy test, an open OFC was performed. If positive test results were obtained, PA diagnosis was confirmed, and the children were eligible for inclusion. We excluded patients with only local reactions to peanuts (e.g., oral allergy syndrome), as this may reflect pollen-related cross-reactivity rather than primary peanut allergy. We also excluded those not consenting to blood sampling.

Venous blood samples were drawn during variable time points after the index reaction. No samples were collected during acute reactions or hospital admissions. The severity of PA was determined based on the most severe reaction encountered and graded according to the 2024 World Allergy Organization (WAO) updated grading system for systemic allergic reactions (10). Isolated systemic cutaneous reactions (WAO grade 1) were defined by the presence of cutaneous allergic reaction within 2 h of peanut consumption with urticaria, pruritus, and/or angioedema, excluding reactions at the contact site with peanut. Patients with at least moderate symptoms and more than one organ involvement were classified into the higher severity cohort (WAO grades 2–5). In patients without a history of life-threatening reactions (WAO grade ≥4) and a peanut sIgE ≤ 25 IU/mL, we offered an open OFC upon obtaining approval by the guardians of these patients. The OFC consisted of serial ingestions of peanut protein under medical supervision every 30 min, starting with 0.25 g of protein and doubling the dose until either signs of a type I hypersensitivity appeared or a final dose of 4 g of peanuts in children <5 years and 8 g in older children was reached.

### 2.2 Outcomes

The primary goal was to compare the clinical markers of peanut reaction severity to differentiate between cutaneous and anaphylactic reactions. The secondary goal was to determine the rate and predictors of spontaneous PA resolution, determined through negative OFC.

Demographic and clinical data were obtained from patients’ medical records and an interview. Data on family history of atopy and comorbid conditions [atopic dermatitis (AD), allergic rhinitis, asthma, inhalant, and other food allergies] were collected. A detailed history on the first reaction to peanut and subsequent reactions was obtained, including the use of an epinephrine autoinjector. The age at presentation of PA was recorded.

Skin-prick testing was conducted on the volar side of the forearm using the peanut antigen–containing solution (Lofarma SpA, Milan, Italy), a positive control (histamine dihydrochloride 10 mg/mL), and a negative control (diluent solution). The wheal diameter was recorded 15 min after prick testing. The test was deemed positive if the peanut was at least 3 mm larger than the negative control. Serum IgE and peanut sIgE, as also sIgE to recombinant antigens Ara h 1, Ara h 2, Ara h 3, Ara h 6, Ara h 8, and Ara h 9 were measured. In addition, sIgE to rye, soy, sesame, hazelnut, walnut, cashew, and pistachio were measured to assess cosensitization. Serum sIgE were measured using the Pharmacia ImmunoCap method (Uppsala, Sweden). A cutoff value ≥0.35 IU/mL confirmed sensitization to the allergen of interest.

A basophil activation test was performed as previously described (11, 12). Briefly, whole blood samples were incubated with peanut extract (Bühlmann Laboratories AG, Switzerland) at concentrations of 0.33, 3.33, and 33.3 ng/mL for 15 min at 37 °C. For controls, cells were incubated with stimulation buffer (negative control) or with 0.55 μg/mL of anti-FcεRI monoclonal antibody (Bühlmann Laboratories AG, Switzerland) as a positive control. Degranulation was stopped by placing the samples on ice, followed by adding CD123-PE, HLA-DR-PerCP, and CD63-FITC-labeled antibodies (BD Biosciences, NJ, USA) and incubated for 20 min. The samples were then lysed, washed, and analyzed using a DxFLEX flow cytometer. Flow cytometry data were analyzed using FlowJo software (version 10.8.1). Diagnostic parameters were calculated for all three mentioned allergen concentrations, and an area under the curve (AUC) analysis of CD63 activation was also performed.

### 2.3 Statistical analysis

A statistical analysis was performed using GraphPad Prism 10 software. Descriptive statistics and tests of normality were performed. The Mann–Whitney U test was performed to compare the ranks of continuous variables (age at last visit, number of food and inhalant allergens sensitized to age at first reaction to peanut; number of reactions to peanut; wheal diameter at SPT to peanut, serum IgE, and peanut sIgE; sIgE to Ara h 1, Ara h 2, Ara h 3, Ara h 6, Ara h 8, and Ara h 9, sIgE to rye, sesame, soy, hazelnut, walnut, pistachio, and cashew; BAT reactivity at 33.3, 3.33, and 0.33 ng/mL; and BAT AUC) between patients with cutaneous and anaphylactic reactions. Linear regression models were fitted using ordinary least squares on complete cases to model the outcome (allergy severity and tolerance to peanut) as a function of biomarker levels, adjusting for age, total IgE, and number of food allergens, as these variables reflect overall allergic sensitization. Model assumptions were assessed using residual plots and Q–Q plots. No major violations were observed. Fisher's exact test was used to compare results in contingency tables. A receiver-operator-characteristic (ROC) curve analysis was used to determine the optimal cutoff values for distinguishing between systemic cutaneous reactions and PA-induced anaphylaxis. The Hanley–McNeil method was used to compare ROC performance. Absolute Cohen's *d* values were calculated to evaluate effect sizes.

### 2.4 Ethical approval

The study was approved by the Ethics Committee of the University Medical Centre Maribor (UKC-MB-KME-25/20). The research was performed in accordance with the Helsinki Declaration of 1975, as revised in Edinburgh in 2000. A written informed consent form was obtained prior to study inclusion, and a separate form was obtained prior to OFC performance.

## 3 Results

### 3.1 Sociodemographic and clinical characteristics of children with systemic cutaneous reactions and peanut allergy–induced anaphylaxis

#### 3.1.1 Family history and sociodemographic characteristics, and coexistent type 2 allergic disorders

**Figure 1:**
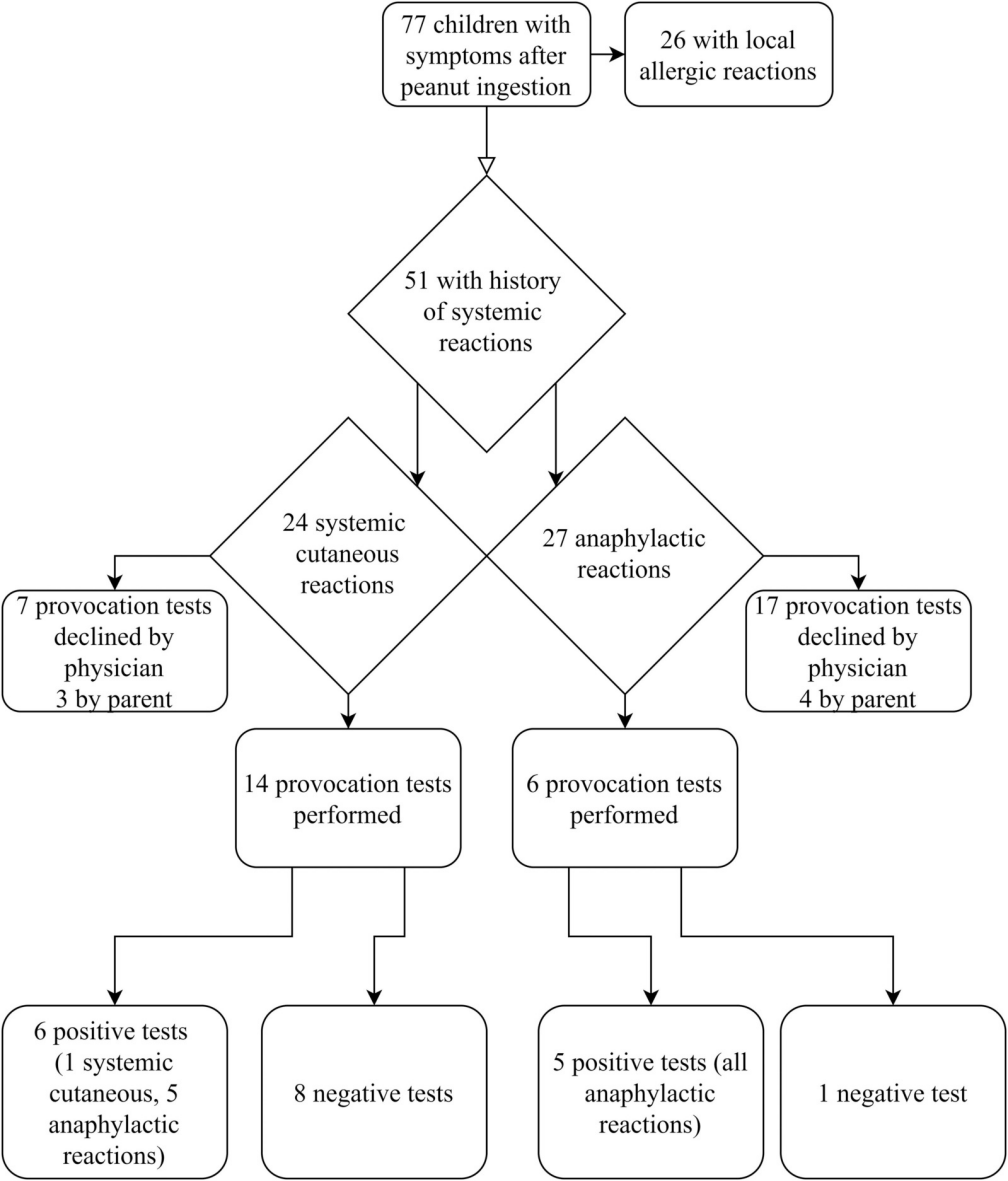
A flowchart of the patient sampling process and peanut oral food challenge results.

Out of 77 children screened, we excluded 26 children with local symptoms after they came into contact with peanuts. A total of 24 children with systemic cutaneous reactions (11 females) and 27 with anaphylactic reactions (7 females) to peanuts were included. The median age of children with systemic cutaneous reactions (72 months) and with anaphylactic reactions (96 months) did not differ (*p* = 0.13). All children were of Caucasian origin. Venous blood was collected from all children. In 16 of 51 children who did not consent to the performance of SPT on the day of venous blood draw, the most recent SPT wheal diameters from previous outpatient visits were recorded. BAT was not successful due to absent basophil response to FcεRI in 3 of 51 patients. Figure 1 provides details on patient selection and Table 1 provides the demographic and clinical characteristics of patients with systemic cutaneous and anaphylactic reactions to peanut.

**Table 1:** Demographic and clinical characteristics of patients with systemic cutaneous and anaphylactic reactions to peanut.

Patient characteristic	Systemic cutaneous reaction, *n* = 24 (95% CI)	Anaphylactic reaction, *n* = 27 (95% CI)	*P*-value
Age (years, median)	6.3 (5.3–10)	8 (6.4–10)	0.130
Female sex (%)	11 (45%)	7 (25%)	0.130
Parental education level	High school: 8 (33%), higher professional school: 1 (4%), bachelors’ and master's degree: 14 (58%), PhD: 1 (4%)	High school: 3 (11%), higher professional school: 5 (18%), bachelors’ and master's degree: 18 (66%), PhD: 1 (3%)	0.500
Family history of food allergy	5 (20%)	6 (22%)	1
peanut allergy	1 (4%)	2 (7%)	1
Family history of allergy to airborne allergens	16 (66%)	17 (63%)	1
Maternal peanut consumption during pregnancy	17 (71%)	22 (81%)	0.500
Breastfeeding duration	Less than 1 month: 2 (8%), 1–3 months: 6 (25%), 4–6 months: 9 (37%), 7–11 months: 4 (16%), 12 months or more: 3 (12%)	Less than 1 month: 2 (7%), 1–3 months: 5 (18%), 4–6 months: 10 (37%), 7–11 months: 8 (29%), 12 months or more: 2 (7%)	0.640
Ownership of household pets	13 (54%)	17 (63%)	0.578
Atopic dermatitis	19 (79%)	22 (81%)	1
Asthma/viral-induced wheeze	3 (13%)	8 (30%)	0.182
Allergic rhinitis	2 (8%)	4 (15%)	0.671
Allergy to other food allergens	8 (33%)	16 (59%)	0.093
to other nuts	5 (21%)	10 (37%)	0.235
Number of food allergens to which the children were allergic	1 (1–2)	2 (1–3)	**0**.**043**
Allergy to airborne allergens	14 (58%)	19 (70%)	0.396
Number of inhalant allergens to which the children were allergic	1 (0–2)	2 (0–3)	**0**.**049**
Age at first reaction	1.5 (1.5–2)	2 (1.5–3)	0.300
Type of exposure to peanut at first reaction	Ingestion: 21 (88%), cutaneous: 3 (13%)	Ingestion: 22 (81%), cutaneous: 2 (7%), inhalation: 3 (11%)	0.496
EpiPen prescription after first reaction	11 (46%)	24 (89%)	**0**.**002**
Avoidance of traces of peanut protein	19 (79%)	24 (89%)	0.451
Number of allergic reactions to peanut	1 (1–2)	2 (1–3)	0.100
EpiPen use by patient/guardian	2 (8%)	1 (4%)	0.596
Allergic reactions to peanut at home	9 (38%)	10 (37%)	1.0
outside of home	3 (13%)	11 (41%)	**0**.**031**
Allergic reactions without evident allergen	5 (21%)	8 (30%)	0.534

Parents of both groups had a similar degree of education. One-fifth of children had a family history of FA and two-third a family history of allergy to airborne allergens. A total of 7% and 12% of children with cutaneous reactions and anaphylactic phenotypes, respectively, were breastfed for at least 12 months (*p* = 0.64). AD was the most prevalent comorbidity present in four-fifth of patients. Allergy to other food allergens was present in 33% of children with cutaneous reactions and in 59% of children with PA-induced anaphylaxis (*p* = 0.09). Coexistent nut allergy was present in 21% and 37% of children with cutaneous reactions and anaphylactic PA phenotype, respectively (*p* = 0.235). Children with PA-induced anaphylaxis were allergic to a median of one other food allergen besides peanut, as opposed to children with cutaneous PA who were mostly monosensitized to peanut (*p* = 0.043). In the anaphylaxis group, children were sensitized to a median of two inhalant allergens and in the cutaneous group to one inhalant allergen (*p* = 0.049).

#### 3.1.2 History of reactions to peanut

The first reaction to peanuts occurred at a median of 18 months in patients with cutaneous reactions and 24 months in patients with anaphylactic reactions (*p* = 0.30). In 12% of patients with cutaneous reactions and 19% of patients with PA-induced anaphylaxis, reactions occurred after non-oral allergen contact. An EpiPen was prescribed after the first reaction in 46% and 89% of patients with cutaneous and anaphylactic reactions, respectively (*p* = 0.002), and 79% (cutaneous phenotype) and 89% (PA-induced anaphylaxis) of patients started avoiding traces of peanut (*p* = 0.451). Patients with anaphylaxis experienced a median of two reactions and patients with systemic cutaneous a single reaction to peanut (*p* = 0.10), but in only 1/16 patients with anaphylaxis to peanut EpiPen was used after a recurrent anaphylactic episode. Allergic reactions occurred outside of home more frequently in the anaphylactic phenotype (*p* = 0.031). A total of 21% of children with cutaneous PA and 30% of children with PA-induced anaphylaxis (*p* = 0.53) experienced systemic allergic reactions to an unknown trigger.

#### 3.1.3 Results of diagnostic tests of children with systemic cutaneous and anaphylactic reactions to peanut

In SPT testing, children with PA-induced anaphylaxis demonstrated greater wheal diameters than children with systemic cutaneous PA (9 vs. 5 mm, *p* = 0.008). Using Benjamini–Hochberg correction, peanut sIgE levels were at median higher in the anaphylactic phenotype (peanut sIgE, 100 vs. 4.5 IU/mL, *p* = 0.001). Median serum-specific sIgE levels to Ara h 1 (13.2 vs. 0.1 IU/mL, *p* < 0.001), Ara h 2 (61.8 vs. 1.0 IU/mL, *p* = 0.001), Ara h 3 (2.3 vs. 0.1 IU/mL, *p* = 0.001), Ara h 6 (34.5 vs. 1.5 IU/mL, *p* = 0.002), and Ara h 8 (5.6 vs. 0.1 IU/mL, *p* = 0.015) but not to Ara h 9 (0.1 vs. 0.1 IU/mL, *p* = 0.06) were higher in the anaphylactic PA phenotype. Ara h 2 values ≥0.35 IU/mL were present in 67% and 85% of children with cutaneous and anaphylactic reaction phenotypes, respectively.

Upon BAT, basophil reactivity was higher in the anaphylactic phenotype at concentrations of 33.3 ng/mL (78% vs. 19%, *p* = 0.003) and 3.3 ng/mL (26% vs. 2%, *p* = 0.012) but not at a concentration of 0.3 ng/mL (2% vs. 1%, *p* = 0.073). A BAT AUC analysis showed significantly higher values in the anaphylactic phenotype (AUC: 1,678 vs. 344, *p* = 0.004). After adjusting for age, total IgE, and number of food allergens to which the children were allergic, SPT wheal diameter (*p* = 0.037); peanut sIgE (*p* = 0.036); IgE to Ara h 1 (*p* = 0.021), Ara h 2 (*p* = 0.041), and Ara h 6 (*p* = 0.030); BAT at 33.3 ng/mL (*p* = 0.039); and BAT AUC values (*p* = 0.040) remained significantly different between groups.

An ROC curve analysis showed the best results (AUC ≥ 0.75) for SPT wheal diameter (AUC: 0.75, *p* = 0.009, 95% CI: 0.59–0.91, cutoff 7 mm); peanut sIgE (AUC: 0.77, *p* = 0.001, 95% CI: 0.63–0.90, cutoff 24 IU/mL); IgE to Ara h 1 (AUC: 0.80, *p* < 0.001, 95% CI: 0.68–0.93, cutoff 0.37 IU/mL), Ara h 2 (AUC: 0.75, *p* = 0.002, 95% CI: 0.63–0.89, cutoff 1.73 IU/mL), Ara h 3 (AUC: 0.75, *p* = 0.002, 95% CI: 0.62–0.90, cutoff 0.39 IU/mL), and Ara h 6 (AUC: 0.75, *p* = 0.003, 95% CI: 0.61–0.89, cutoff 8.44 IU/mL); BAT at 33.3 ng/mL concentration (AUC: 0.75, *p* = 0.003, 95% CI: 0.58–1.00, cutoff 33%) and 3.3 ng/mL concentration (AUC: 0.71, *p* = 0.014, 95% CI: 0.56–0.86, cutoff 5%); and BAT area under the CD63 response curve analysis (AUC: 0.74, *p* = 0.004, 95% CI: 0.60–0.89, cutoff 535). ROC curve performances of BAT at 33.3 ng/mL in comparison with the ROC performance of SPT and sIgE to Ara h 1, Ara h 2, Ara h 3, and Ara h 6 were not statistically different based on the Hanley–McNeil test (smallest *p* = 0.44). Interestingly, a significant difference was also seen for Ara h 8 (ROC AUC: 0.69, *p* = 0.019, 95% CI: 0.54–0.84, cutoff 1.20 IU/mL). Other allergy tests (IgE Ara h 9 and BAT at 0.3 ng/mL) did not reach statistical significance. With regard to sensitization to other nuts and selected food allergens, children with PA-induced anaphylaxis demonstrated marginally higher sIgE to soy (0.92 vs. 0.17 IU/mL, *p* = 0.024), which was not observed after adjusting for age, total IgE, and food polysensitization. No differences were observed in sIgE to hazelnut, walnut, pistachio, rye, and sesame.

**Figure 2:**
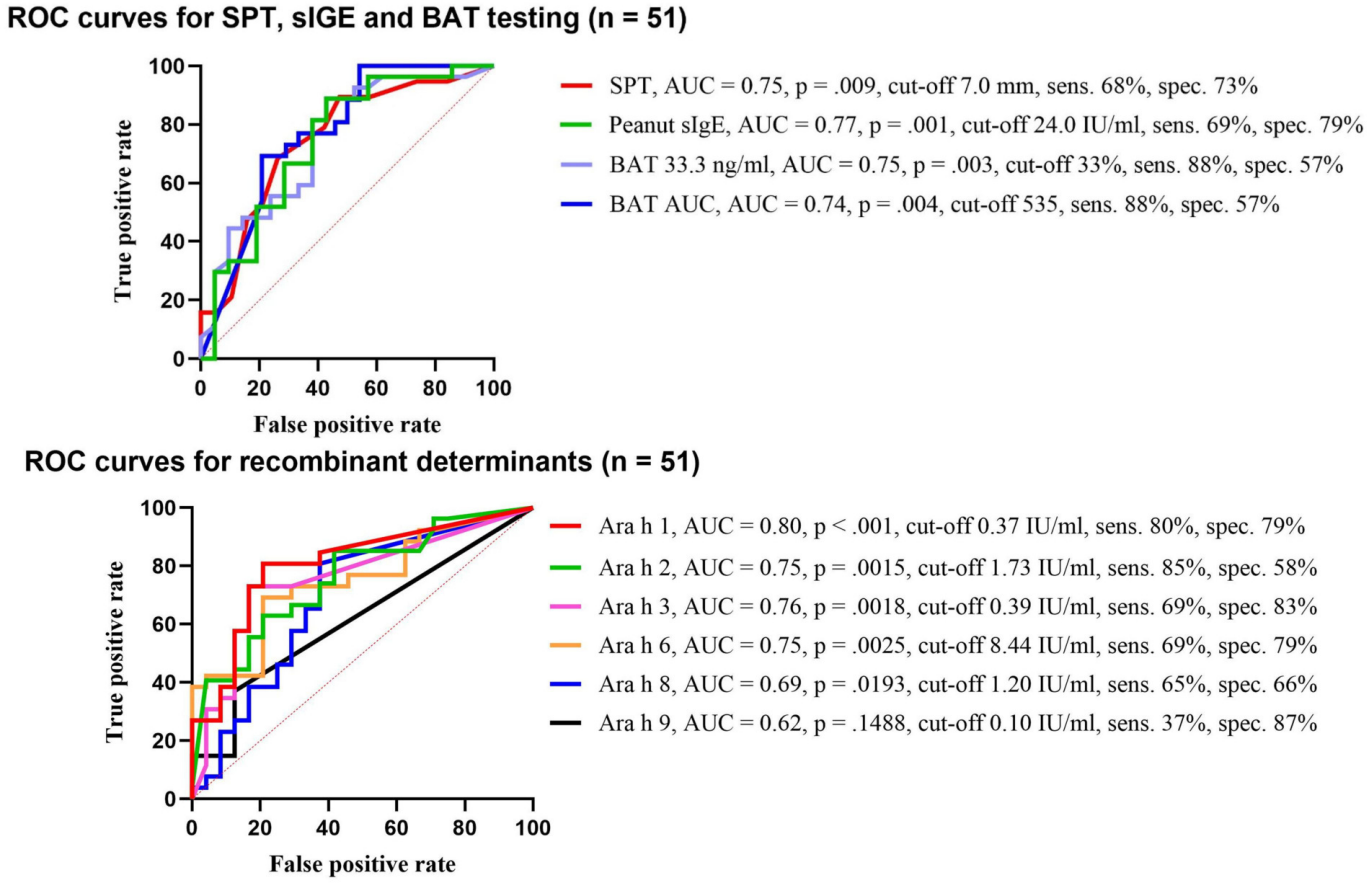
An ROC curve analysis of the skin-prick test wheal diameter, peanut sIgE levels, specific IgE levels to Ara h 1, Ara h 2, Ara h 3, Ara h 6, Ara h 8, and Ara h 9, and BAT results discriminating between cutaneous and anaphylactic reactions to peanut. Sens., sensitivity. Spec., specificity.

Table 2 provides the median values of skin-prick testing, serum-specific IgE, and BAT reactivity to peanut for patients with a history of cutaneous and anaphylactic reactions to peanut. Figure 2 provides ROC curves with sensitivities, specificities, and cutoff values for selected allergy tests.

**Table 2:** Results of skin-prick testing, serum-specific IgE, and BAT reactivity to peanut for patients with a history of cutaneous and anaphylactic reactions to peanut.

Median values of tests	Systemic cutaneous reaction, *n* = 24 (95% CI)	Anaphylactic reaction, *n* = 27 (95% CI)	Cohen's *d*	Unadjusted *P*-value	Regression coefficient *β*	Adjusted *P*-value
SPT wheal size	5 (3–8)	9 (7–10)	0.89	**0**.**008**^a^	3	**0**.**037**
Serum total IgE (IU/mL)	362 (152–943)	774 (382–1,665)	0.55	0.038	178	0.357
Peanut sIgE (IU/mL)	4.5 (1.4–21.5)	100 (20.3–100)	0.87	**0**.**001**^a^	24	**0**.**036**
Serum-specific IgE levels to Ara h 1 (IU/mL)	0.1 (0.1–0.3)	13.2 (1.3–43.5)	0.88	**<0**.**001**^a^	20	**0**.**021**
Serum-specific IgE levels to Ara h 2 (IU/mL)	1.0 (0.17–17.6)	61.8 (5.6–100.0)	0.59	**0**.**001**^a^	23	**0**.**041**
Serum-specific IgE levels to Ara h 3 (IU/mL)	0.1 (0.10–0.1)	2.3 (0.2–31.3)	0.60	**0**.**001**^a^	9	0.24
Serum-specific IgE levels to Ara h 6 (IU/mL)	1.5 (0.10–8.1)	34.5 (6.2–91.4)	0.88	**0**.**002**^a^	23	**0**.**030**
Serum-specific IgE levels to Ara h 8 (IU/mL)	0.1 (0.10–8.1)	5.6 (0.2–26.4)	0.41	**0**.**015**^a^	3	0.670
Serum-specific IgE levels to Ara h 9 (IU/mL)	0.1 (0.1–0.1)	0.1 (0.1–0.14)	0.40	0.060	1	0.713
BAT reactivity to peanut at 33.3 ng/mL	19 (6–75)	78 (50–91)	0.86	**0**.**003**^a^	20	**0**.**039**
BAT reactivity to peanut at 3.3 ng/mL	2 (1–21)	26 (3–65)	0.67	**0**.**012**^a^	14	0.093
BAT reactivity to peanut at 0.3 ng/mL	1 (1–2)	2 (1–5)	0.30	0.073	2	0.313
BAT area under the curve to peanut	344 (108–1,588)	1,678 (766–2,373)	0.85	**0**.**004**^a^	**553**	**0**.**040**
Serum-specific IgE levels to rye (IU/mL)	0.1 (0.1–0.3)	0.1 (0.1–0.18)	0.29	0.914	−1	0.537
Serum-specific IgE levels to soy (IU/mL)	0.17 (0.1–1.16)	0.92 (0.2–7.0)	0.72	**0**.**024**^a^	8	0.177
Serum-specific IgE levels to sesame (IU/mL)	0.35 (0.11–1.74)	0.80 (0.35–2.29)	0.38	0.321	−3	0.666
Serum-specific IgE levels to hazelnut (IU/mL)	0.72 (0.1–4.43)	2.43 (0.85–10.0)	0.52	0.066	2	0.626
Serum-specific IgE levels to walnut (IU/mL)	0.1 (0.1–1.12)	0.14 (0.10–0.44)	0.19	0.815	0	0.976
Serum-specific IgE levels to cashew (IU/mL)	0.1 (0.10–0.13)	0.19 (0.10–1.53)	0.42	0.049	1	0.870
Serum-specific IgE levels to pistachio (IU/mL)	0.27 (0.10–0.65)	0.94 (0.21–2.61)	0.44	0.134	0	0.990

a Marks significant *P*-values after Benjamini–Hochberg correction. *P*-values were adjusted for age, serum total IgE, and number of food allergens to which the children were allergic.

### 3.2 Comparison of sociodemographic and clinical characteristics of children with persistent and resolved peanut allergy

#### 3.2.1 Sociodemographic and clinical factors affecting PA resolution

For 51 children with a history of systemic symptoms after ingestion of peanut, we offered to perform 27 OFCs. In 24 patients with a history of a life-threatening reaction to peanut or peanut sIgE levels ≥25 IU/mL, we withheld provocation testing. A total of 7/27 (26%) caregivers declined provocation testing. We performed 14 OFCs in children with a history of systemic cutaneous PA (58% of all patients) and 6 OFCs in PA-induced anaphylaxis (22% of all patients). Resolution of PA, as defined by negative OFCs, was present in 9/20 OFCs (8 in patients with a history of cutaneous reaction and 1 in a patient with a history of a PA-induced anaphylaxis). One negative OFC was observed in a child with elevated sIgE to Ara h 2 (1.52 IU/mL), and 3/11 necessitated epinephrine application after OFC (with Ara h 2 levels of 23.6, 1.9, and 1.1 IU/mL).

Children in the resolved PA group (*n* = 9) were significantly older than those in the persistent PA group (*n* = 11), with median ages of 10.7 and 5.7 years, respectively (*p* = 0.004). Consistently, the interval since PA diagnosis was significantly longer in children with resolved PA compared with those with persistent PA (median 8.5 vs. 3.0 years, *p* = 0.018). Other factors such as gender, parental education level, family history of food or inhalant allergy, maternal peanut consumption during pregnancy, duration of breastfeeding, atopic comorbidities, age at first reaction, and level of avoidance to peanuts did not significantly differ between groups. Table 3 provides the demographic and clinical characteristics of patients with persistent and resolved PA based on an OFC.

**Table 3:** Demographic and clinical characteristics of patients with persistent and resolved peanut allergy based on an open-label oral food challenge.

Patient characteristic	Persistent, *n* = 11 (95% CI)	Resolved, *n* = 9 (95% CI)	*P*-value
Age (years, median)	5.7 (2.9–9.7)	10.7 (6.6–11.7)	**0**.**004**
Duration since PA diagnosis (years, median)	3.0 (2.5–6.1)	8.5 (4.5–9.5)	**0**.**018**
Female sex (%)	3 (27%)	3 (33%)	1.0
Parental education level	High school: 4 (36%), higher professional school: 1 (9%), bachelors’ and master's degree: 6 (55%)	High school: 3 (33%), higher professional school: 0 (0%), bachelors’ and master's degree: 5 (55%)	0.904
Family history of food allergy	1 (9%)	1 (11%)	1.0
peanut allergy	0	0	1.0
Family history of allergy to airborne allergens	7 (64%)	5 (45%)	1.0
Maternal peanut consumption during pregnancy	10 (91%)	5 (56%)	0.127
Breastfeeding duration	Less than 1 month: 0 (0%), 1–3 months: 3 (27%), 4–6 months: 4 (36%), 7–11 months: 3 (27%), 12 months or more: 1 (9%)	Less than 1 month: 1 (11%), 1–3 months: 2 (22%), 4–6 months: 4 (44%), 7–11 months: 1 (11%), 12 months or more: 1 (11%)	0.630
Ownership of household pets	8 (73%)	6 (67%)	1.0
Atopic dermatitis	9 (82%)	7 (78%)	1.0
Asthma/viral-induced wheeze	1 (9%)	1 (11%)	1.0
Allergic rhinitis	0 (0%)	1 (11%)	0.450
Allergy to other food allergens	4 (36%)	4 (44%)	1.0
to other nuts	3 (27%)	4 (44%)	0.643
Number of food allergens to which the children were allergic	1 (1–2)	1 (1–2)	1.0
Allergy to airborne allergens	4 (36%)	6 (66%)	0.370
Number of inhalant allergens to which the children were allergic	0 (0–2)	2 (0–3)	0.170
Age at first reaction	1.5 (0.75–3.5)	2.0 (1.5–2.75)	0.352
Type of exposure to peanut at first reaction	Ingestion: 9 (82%), cutaneous: 2 (18%)	Ingestion: 9 (100%)	0.479
EpiPen prescription after first reaction	8 (73%)	5 (55%)	0.643
Avoidance of traces of peanut protein	8 (73%)	6 (66%)	1.0
Number of allergic reactions to peanut	2 (1–3)	1 (1–4)	0.98
EpiPen use by patient/guardian	0 (0%)	0 (0%)	1.0
Allergic reactions to peanut at home	5 (45%)	1 (11%)	0.157
outside of home	2 (18%)	3 (33%)	0.617
Allergic reactions without evident allergen	2 (18%)	0 (0%)	0.479

#### 3.2.2 Diagnostic tests for evaluating PA resolution

Upon allergy testing, resolution of PA was associated with lesser SPT wheal diameters (3 vs. 7 mm, *p* = 0.014), lower peanut sIgE levels (0.77 vs. 3.61 IU/mL, *p* = 0.030), and lower levels of sIgE to Ara h 2 (0.10 vs. 1.88 IU/mL, *p* = 0.002) and Ara h 6 (0.1 vs. 1.26 IU/mL, *p* = 0.001) in an unadjusted analysis. No differences were observed between serum total IgE; peanut sIgE to Ara h 1, Ara h 3, Ara h 8, and Ara h 9 levels; and between sIgE levels to rye, soy, sesame, hazelnut, walnut, cashew, and pistachio. BAT reactivity was lower in the resolved group at 33.3 ng/mL (5% vs. 32%, *p* = 0.033) and the AUC analysis (AUC: 78 vs. 531, *p* = 0.027). At 3.3 ng/mL, there was no difference. At a concentration of 0.3 ng/mL, BAT reactivity was 1% in both groups. A total of 12/20 children undergoing OFC had BAT reactivity ≥5%, a cutoff reported by Riggioni et al. (13), and 4/12 of children with BAT reactivity ≥5% proved tolerant to peanut. After adjustment for relevant confounders, peanut sIgE (*p* = 0.033) and IgE to Ara h 2 (*p* = 0.032) and Ara h 6 (*p* = 0.020) remained significantly different between groups, and BAT showed a possible association (*p* = 0.06).

An ROC curve biomarker analysis of persistent and resolved cases showed the best results (AUC > 0.80) for SPT wheal diameter (AUC: 0.90, *p* = 0.016, 95% CI: 0.73–1.00, cutoff 5 mm), IgE to Ara h 2 (AUC: 0.88, *p* = 0.004, 95% CI: 0.72–1.00, cutoff 0.40 IU/mL) and Ara h 6 (AUC: 0.94, *p* = 0.001; 95% CI: 0.82–1.00, cutoff 0.35 IU/mL), and BAT at the highest concentration (AUC: 0.81, *p* = 0.03, 95% CI: 0.58–1.00, cutoff 25%). An ROC of BAT at 33.3 ng/mL had statistically similar performance to SPT, Ara h 2, and Ara h 6 (smallest *p* = 0.22).

Table 4 provides the results of skin-prick testing, serum-specific IgE, and BAT reactivity to peanut for patients with persistent and resolved peanut allergy based on an OFC.

**Table 4:** Results of skin-prick testing, serum-specific IgE, and BAT reactivity to peanut for patients with persistent and resolved peanut allergy based on an open-label oral food challenge.

Median values of tests	Persistent, *n* = 11 (95% CI)	Resolved, *n* = 9 (95% CI)	Cohen's *d*	Unadjusted *P*-value	Regression coefficient *β*	Adjusted *P*-value
SPT wheal size (mm)	7 (4.0–7)	3 (0–4)	1.83	**0**.**014**	−3	0.257
Serum total IgE (IU/mL)	171 (54–513)	729 (152–1,362)	1.1	0.056	376	0.165
Peanut sIgE (IU/mL)	3.61 (1.52–20.3)	0.77 (0.1–5.2)	0.96	**0**.**030**	**−8**	**0**.**033**
Serum-specific IgE levels to Ara h 1 (IU/mL)	0.1 (0.1–0.47)	0.1 (0.10–0.38)	0.03	0.596	0	0.752
Serum-specific IgE levels to Ara h 2 (IU/mL)	1.88 (0.17–12.5)	0.10 (0.1–0.2)	0.88	**0**.**002**^a^	**−5**	**0**.**032**
Serum-specific IgE levels to Ara h 3 (IU/mL)	0.1 (0.1–0.57)	0.1 (0.1–0.1)	0.67	0.479	0	0.256
Serum-specific IgE levels to Ara h 6 (IU/mL)	1.26 (0.57–8.38)	0.1 (0.1–0.1)	1.19	**0**.**001**^a^	**−4**	**0**.**020**
Serum-specific IgE levels to Ara h 8 (IU/mL)	2.14 (0.10–20.9)	0.1 (0.1–28.5)	0.16	0.815	0	0.986
Serum-specific IgE levels to Ara h 9 (IU/mL)	0.1 (0.1–0.1)	0.1 (0.1–0.33)	0.35	0.45	0	0.741
BAT reactivity to peanut at 33.3 ng/mL	32 (7–50)	5 (1–18)	1.21	**0**.**033**	**−23**	0.060
BAT reactivity to peanut at 3.3 ng/mL	1 (1–3)	1 (0–6)	0.29	0.165	−1	0.445
BAT reactivity to peanut at 0.3 ng/mL	1 (0–1)	1 (1–4)	1.08	**0**.**036**	**1**	0.342
BAT area under the curve to peanut	531 (121–765)	78 (16–345)	1.16	**0**.**027**	−366	0.066
Serum-specific IgE levels to rye (IU/mL)	0.10 (0.10–0.38)	0.1 (0.10–1.21)	0.52	0.976	1	0.996
Serum-specific IgE levels to soy (IU/mL)	0.19 (0.10–0.26)	0.15 (0.10–1.14)	0.48	0.623	0	0.487
Serum-specific IgE levels to sesame (IU/mL)	0.35 (0.10–1.02)	0.19 (0.10–8.28)	0.41	0.840	2	0.501
Serum-specific IgE levels to hazelnut (IU/mL)	1.60 (0.10–5.50)	1.79 (0.10–21.10)	0.47	0.807	3	0.635
Serum-specific IgE levels to walnut (IU/mL)	0.10 (0.10–0.32)	0.10 (0.10–1.12)	0.46	0.560	1	0.859
Serum-specific IgE levels to cashew (IU/mL)	0.10 (0.10–0.10)	0.10 (0.10–0.12)	0.53	0.074	0	0.576
Serum-specific IgE levels to pistachio (IU/mL)	0.21 (0.10–0.90)	0.21 (0.10–3.30)	0.38	0.457	0	0.999

a Marks significant *P*-values after adjustment using the Benjamini–Hochberg procedure. *P*-values were adjusted for age, serum total IgE, and number of food allergens to which the children were allergic.

## 4 Discussion

### 4.1 Risk factors for severe systemic reactions to peanut

With regard to factors associated with peanut reaction severity, only sensitization to multiple food and inhalational allergens was associated with more severe allergic phenotype. Factors such as family history of FA and inhalant allergy, gender, parental education level, duration of breastfeeding, and the presence of allergic upper and lower airway disease and AD were not associated with reaction severity. This contrasts with population-based adult data in which the number of allergic diseases was positively associated with anaphylaxis, with the highest risk in patients with FA and asthma (14). However, a meta-analysis of 88 studies found no consistent evidence that the mere presence of asthma, diagnosed in up to 50% of food-allergic individuals, is associated with increased severity of food-induced anaphylaxis. Based on a meta-analysis, a history of prior anaphylaxis and the degree of IgE sensitization (SPT, peanut sIgE levels) are poor predictors of anaphylaxis (15).

### 4.2 Risk factors for the development of PA

Previously reported risk factors for the presence of PA (but not reaction severity) include a family history of peanut allergy, the presence of AD and allergic airway disease, allergies to other food and airborne allergens, and late introduction of peanut (16–20). In a high-risk infant cohort, Sicherer et al. identified associations of PA with a lack of breastfeeding, younger age, and greater sIgE to peanut and Ara h 2 (21).

In line with the literature, a positive family history of FA, PA, and allergy to airborne allergens was observed in our results, as were the presence of allergic airway disease and AD. Up to two-third of children exhibited allergies to other food allergens and up to 37% to other nuts. We have already reported that Slovene caregivers introduce peanuts late, at a median of 12 months (22), representing a risk factor for PA development (23, 24).

### 4.3 Capability of the allergy test to distinguish between cutaneous and anaphylactic reactions to peanut

Allergy testing modalities such as SPT, specific sIgE to whole peanut, component-resolved diagnostics, and BAT offer a greater insight into the sensitization profile of patients. However, PA severity is influenced by several factors beyond the degree of sensitization (6). In our cohort, we observed greater values of SPT and peanut sIgE levels in patients with anaphylactic reactions, as well as higher sIgE to major peanut protein antigens Ara h 1, Ara h 2, and Ara h 6 after adjustment for age, total IgE, and the number of food allergens to which they were allergic.

Datema et al. (25) estimated the risk of severe PA using clinical background and IgE sensitization profiles and showed that female sex, age at onset of PA, symptoms elicited by skin contact with peanut, family atopy, AD, house dust mite, and latex allergy were independently associated with severe PA, and birch pollen allergy was associated with mild-to-moderate PA. The observation by Datema et al. (25) of higher levels of Ara h 1, Ara h 2, and Ara h 6 in the anaphylactic phenotype was also established in our cohort. However, we did not observe any difference in phenotype severity regarding sex, family atopy, age of onset of PA, and AD presence. Because of the nature of the design, we excluded cross-reactive patients with birch pollen allergy.

Santos et al. (6) reported that the SPT (cutoff 8 mm), whole peanut sIgE (cutoff 5 kU/L), and Ara h 2 sIgE (cutoff 1.4 kU/L) levels also demonstrated 100% sensitivity with slightly lower specificity (90%–93%) in predicting the severity of reactions. Ojaniemi et al. (26) reported that Ara h 2 sIgE were associated with a positive outcome in peanut OFC but were not a reliable predictor of reaction severity. In our study, the cutoff values of 7 mm for SPT and 1.73 IU/mL for sIgE to Ara h 2 are comparable to reported literature (6). sIgE levels ≥24 IU/mL were correlated with anaphylactic reactions, a threshold fivefold higher than the reported 4.3 kU/L median cutoff for the diagnosis of PA (13).

The performance of Ara h 6 at cutoff levels of 8.44 IU/mL was very similar to that of Ara h 2 to distinguish between systemic cutaneous and more severe reactions. This is related to the structural composition of the Ara h 6 protein, which shares approximately 60% sequence identity and multiple epitopes with Ara h 2 (27). The association of Ara h 6 sensitization with severe reactions to peanuts in children has been described by Kukkonen et al. (28), and sIgE to Ara h 6 in combination with Ara h2 identified all severe reactions at low doses. Similarly, based on a proteomic analysis of sera of pediatric patients, sIgE to Ara h 2 and Ara h 6 significantly correlated with PA severity (29).

Interestingly, sIgE to Ara h 1 achieved robust diagnostic performance at a cutoff value of 0.39 IU/mL with 80% sensitivity and 79% specificity, with values ≥0.35 IU/mL being present in 33% and 37% of children with cutaneous and anaphylactic reaction phenotypes, respectively. Based on the literature, sIgE to Ara h 1 are associated with more severe systemic reactions at OFC in patients with cosensitization to other storage proteins (30). In the EuroPrevall study, sensitization to Ara h 1 and Ara h 2 was exclusively observed in early-onset PA (31).

Santos et al. (6) also reported that BAT identified any anaphylactic with high sensitivity (100%) and specificity (97%) at a cutoff value of 48%. In our cohort, using the highest concentration of peanut extract, BAT reactivity was correlated with anaphylactic reactions and at a cutoff value of 33% reactivity achieved 88% sensitivity and 57% specificity.

### 4.4 Capability of the allergy test to predict negative OFC and tolerance to peanut

In our study, PA resolution was observed in nine patients and was associated with greater age at OFC, lower peanut sIgE, and sIgE to Ara h 2 and Ara h 6 levels after adjusting for age, total IgE, and the number of food allergens to which they were allergic. Lower BAT reactivity at the highest concentration and calculated BAT AUC values showed a trend toward negative OFC outcomes but did not reach statistical significance (*p* = 0.06). Combining 11 patients who developed symptoms at OFCs with 24 patients with a history of life-threatening reactions or peanut sIgE levels ≥25 IU/mL not undergoing OFCs, whom we considered likely allergic, tolerance to peanut was achieved in 9 out of 44 children (20%).

Foong et al. (32) reported biomarkers of PA over time and found that children with resolved PA demonstrated lesser SPT, sIgE, and Ara h 2 levels, similar to our findings. Skolnick et al. reported that PA was outgrown in approximately 21% of patients (33). Parker et al. (34) reported that one-third of infant PA resolved by 10 years of age and correlated with decreasing sIgE and sIgG4 to peanut and Ara h 2 over time. We observed a similar age of patients who achieved tolerance, as also lower Ara h 2 sIgE levels in the peanut tolerant cohort.

With regard to BAT to evaluate tolerance, Santos et al. reported that basophil sensitivity was associated with the threshold of allergic reactions to peanut and could predict reactivity to 0.1 g or less of peanut protein (35). It was shown that BAT could reduce the need for OFC after equivocal SPT or Ara h 2 sIgE testing (11). In our study, 12 of 20 children undergoing OFC had BAT reactivity ≥5%, a “cutoff” reported by Riggioni et al. (13), and 4 of 12 children with BAT reactivity ≥5% proved tolerant to peanut.

### 4.5 Clinical applications

When managing patients with established PA and a history of systemic reactions, those with sensitization to multiple food and inhalant allergens, in addition to traditional risk factors such as poorly controlled asthma and a history of anaphylaxis, may require more extensive counseling on epinephrine autoinjector use. As reactions experienced outside of home were frequently of greater severity, these findings reinforce the necessity of maintaining epinephrine autoinjector availability. In children with systemic reactions to peanut, SPT wheal diameters ≥7 mm, sIgE to peanut 24 IU/mL, and elevated levels of sIgE to Ara h 1, Ara h 2, and Ara h 6 above reported cutoffs are associated with severe reactions. Evaluating these biomarkers may help reduce the need for OFCs in selected cases. In patients with equivocal results, BAT reactivity ≥33% also correlates with a severe phenotype. When assessing resolution, declining levels of peanut sIgE, Ara h 2, and Ara h 6 are informative.

### 4.6 Study limitations

Our study is limited by a relatively small cohort size, which may have affected the power of statistical tests. However, all individuals had detailed measurements related to peanut sensitization, including SPT results, sIgE level, peanut recombinant profiles, and BAT results. Furthermore, in 60% of the children we did not perform an OFC; the main reason for this was the relatively high sensitization levels, and in some cases, non-compliance from parents. Comparing children without OFC, only 3 of 10 of children with cutaneous and 4 of 21 of PA-induced anaphylaxis cases demonstrated peanut sIgE <25 kU/L, which is predictive of PA. Although this approach preserves clinical relevance, where OFCs are selectively performed based on risk assessment, it introduces selection and verification bias and may lead to an overestimation of the diagnostic performance of the evaluated biomarkers. Finally, the generalizability of the findings is limited, as the cohort is relatively small and derived from a single geographic region with a homogeneous population.

## Key message

This study highlights that high sensitization levels measured by peanut SPT; sIgE to peanut, Ara h 1, Ara h 2, and Ara h 6; or BAT are similarly and significantly associated with the risk of severe peanut allergy. However, it was found that only peanut sIgE, Ara h 2, and Ara h 6 were also associated with PA resolution. In a limited sample of pediatric patients, peanut sIgE, Ara h 2, and Ara h 6 showed moderate diagnostic performance regarding severity risk and PA resolution. Those observations emphasize the importance and high clinical utility of cost-effective skin testing or component-resolved diagnostics in comparison with demanding and complicated cellular tests.

## Data Availability

The raw data supporting the conclusions of this article will be made available by the authors without undue reservation.
